# Comparing Microsporidia-targeting primers for environmental DNA sequencing[Fn FN1]

**DOI:** 10.1051/parasite/2023056

**Published:** 2023-11-28

**Authors:** Annemie Doliwa, Daniel Grabner, Bernd Sures, Micah Dunthorn

**Affiliations:** 1 Aquatic Ecology and Centre for Water and Environmental Research (ZWU), University of Duisburg-Essen Universitätsstrasse 5 45141 Essen Germany; 2 Research Center One Health Ruhr, Research Alliance Ruhr, University of Duisburg-Essen 45141 Essen Germany; 3 Natural History Museum, University of Oslo 0562 Oslo Norway

**Keywords:** Barcoding, Diversity, Metabarcoding, Microsporidia, Parasites, Protists

## Abstract

Metabarcoding is a powerful tool to detect classical, and well-known “long-branch” Microsporidia in environmental samples. Several primer pairs were developed to target these unique microbial parasites, the majority of which remain undetected when using general metabarcoding primers. Most of these Microsporidia-targeting primer pairs amplify fragments of different length of the small subunit ribosomal RNA (SSU-rRNA) gene. However, we lack a broad comparison of the efficacy of those primers. Here, we conducted *in silico* PCRs with three short-read (which amplify a few-hundred base pairs) and two long-read (which amplify over a thousand base pairs) metabarcoding primer pairs on a variety of publicly available Microsporidia *sensu lato* SSU-rRNA gene sequences to test which primers capture most of the Microsporidia diversity. Our results indicate that the primer pairs do result in slight differences in inferred richness. Furthermore, some of the reverse primers are also able to bind to microsporidian subtaxa beyond the classical Microsporidia, which include the metchnikovellidan *Amphiamblys* spp., the chytridiopsid *Chytridiopsis typographi* and the “short-branch” microsporidian *Mitosporidium daphniae*.

## Abstract (fr)

Le métabarcoding est un outil puissant pour détecter les microsporidies classiques et bien connues à « longues branches » dans les échantillons environnementaux. Plusieurs paires d’amorces ont été développées pour cibler ces parasites microscopiques exceptionnels, dont la majorité restent indétectables lors de l’utilisation d’amorces générales de métabarcoding. La plupart de ces paires d’amorces ciblant les microsporidies amplifient des fragments de différentes longueurs du gène de la petite sous-unité de l’ARN ribosomal (SSU-rRNA). Cependant, nous manquons d’une comparaison générale de l’efficacité de ces amorces. Ici, pour tester quelles amorces capturent la plus grande partie de la diversité des microsporidies, nous avons réalisé des PCR *in silico* avec trois paires d’amorces de métabarcoding à lecture courte (qui amplifient quelques centaines de paires de bases) et deux paires d’amorces de métabarcoding à lecture longue (qui amplifient plus d’un millier de bases), sur une variété de séquences du gène SSU-rRNA de Microsporidia *sensu lato* accessibles au public. Nos résultats indiquent que les paires d’amorces entraînent de légères différences dans la richesse déduite. En outre, certaines des amorces inverses sont également capables de se lier à des sous-taxons de microsporidies au-delà des Microsporidia classiques, notamment les Metchnikovellidae *Amphiamblys* spp., le Chytridiopsida *Chytridiopsis typographi* et la microsporidie à « branches courtes » *Mitosporidium daphniae*.

## Introduction

Metabarcoding is a powerful method that uses primers to amplify informative gene regions to uncover microbial diversity in different aquatic, terrestrial, and host environments [3, 23, 26]. Identifying suitable primers for studies of environmental protist diversity is problematic, due to the primers not being able to amplify the full spectrum of species in the desired taxon [19, 26]. For example, general small subunit rRNA (SSU-rRNA) primers that were designed to amplify most eukaryotes are not able to bind to many taxa that have high rates of evolution, such as parasites [27, 30, 36]. One such parasitic taxon that often remains undetected by general eukaryotic metabarcoding primer pairs is the Microsporidia [36].

Microsporidia *sensu lato* contains highly diverse microbial eukaryotes that are increasingly important parasites of many metazoans and some protists [7]. Their diversity comprises the “long-branch” microsporidians including the classical microsporidians, as well as chytridiopsids and metchnikovellids. Their diversity also includes several lineages of “short-branch” microsporidians that display some long-branch-like characteristics, but have a lower number of derived characteristics and lower rates of nucleotide substitutions [4]. While general protistan metabarcoding primers targeting the V4 region of the SSU-rRNA gene can amplify short-branch microsporidians [4, 8], more specific primers are necessary to amplify the majority of classical microsporidians. Some microsporidian-specific primers target small fragments of the SSU-rRNA gene (e.g., [25, 28, 36]); these primers amplify a couple to a few hundred base pairs of DNA, which can be sequenced using short-read sequencing platforms such as Illumina. Other microsporidian-specific primers target much longer fragments of the SSU-rRNA gene (e.g., [11, 29]); these primers amplify sequences of over a thousand base pairs, which require sequencing using long-read sequencing platforms such as PacBio or MinION. However, there has been little work done to compare the efficacy of these primers to uncover microsporidians from environmental samples and host tissues (e.g., [25]).

In this study, we conducted *in silico* amplifications with a selection of microsporidian-targeting primers on a publicly available reference data set consisting of microsporidian SSU-rRNA gene sequences. We compared the extent to which the detectable microsporidian richness differs between the primer pairs and which subtaxa each individual primer will preferably amplify. Our results indicate slight variations in the inferred richness depending on the primer combination used. Further, some primers targeted microsporidian subtaxa beyond the classical microsporidians.

## Material and methods

### Primers and sequences

The two forward and five reverse primers listed in Table 1 were tested individually and in commonly used combinations. These combinations comprise primer pairs resulting in either short reads (CM-V5F/CM-V5R, V1F/micuni3R, and V1F/530R) or long reads (V1F/1342R, V1F/1492R), and have been widely used to amplify classical microsporidians (see Table S1 for example literature). Full length SSU-rRNA gene sequences were taken from a reference alignment published by Dubuffet et al. [9]. To reduce the risk of false negatives due to missing sequence information, their alignment was checked by eye in Aliview v1.27 [14] for signature sequences corresponding to the potential binding regions of V1F and 1492R. Gaps within the selected sequences were removed in R v.4.1.0 [21] integrated in RStudio v.2022.02.3+492 [22] using the del.gaps() function of ape v5.5 [18].

**Table 1:** Primers analyzed in this study. These primers were also tested as the following standard primer pair combinations: CM-V5F/CM-V5R, V1F/micuni3R, V1F/530R, V1F/1342R, and V1F/1492R.

Primer	Sequence	Reference
CM-V5F	5′-GAT TAG ANA CCN NNG TAG TTC-3′	Trzebny et al. [25]
V1F	5′-CAC CAG GTT GAT TCT GCC TGA C-3′	Zhu et al. [37]
530R	5′-CCG CGG CKG CTG GCA C-3′	Baker et al. [2]
1342R	5′-ACG GGC GGT GTG TAC AAA GAA CAG-3′	McClymont et al. [15]
1492R	5′-GGT TAC CTT GTT ACG ACT T-3′	Weiss et al. [29]
CM-V5R	5′-TAA NCA GCA CAM TCC ACT C-3′	Trzebny et al. [25]
Micuni3R	5′-ATT ACC GCG GMT GCT GGC AC-3′	Weigand et al. [28]

### Analyses

We predicted the primer-template sequence bindings in Geneious Prime v2022.0.1 (Biomatters), using the function “Test with saved primers…” (Primer design uses a modified version of Primer3 v2.3.7), and set the maximum number of mismatches to zero, one, two and three. For primer pair testing, the “pairs only” function was activated, which considers the chosen primers only as inward-directed primer pairs. It should be noted that Geneious may treat “N” as any nucleotide, and therefore an “N” may not result in a mismatch when compared to any nucleotide. A supplemental figure (File S2) depicting binding positions and mismatches and a virtual gel image (Figure S2) were created in Geneious. Output tables were edited manually and analyzed in R using the packages ggforce v0.3.3 [20], ggpubr v0.4.0 [13], ggtext v0.1.2 [35], plyr v1.8.6 [32], RColorBrewer v1.1-2 [17], readxl v1.3.1 [34], and tidyverse v1.3.1 [33] including ggplot2 v.3.4.2 [31]. The taxonomic assignment was updated according to Bojko et al. [5]. We filtered out off-target predicted primer bindings from the main analyses and figures to improve data visualization and avoid multiple counts of affected microsporidians. The excluded off-targets are provided separately in the supplements.

## Results and discussion

After curating the alignment of Dubuffet et al. [9], we kept 130 sequences (File S1, Table S2). We included 129 long-branch microsporidians, of which 126 were classical microsporidians: Amblyosporida, 12 sequences; Caudosporida, two sequences; Enterocytozoonida, 21 sequences; Glugeida, 36 sequences; Glugeida+, two sequences; Neopereziida, 16 sequences; Nosematida, 33 sequences; Ovavesiculida, four sequences. Further included microsporidians were: metchnikovellids, two sequences; chytridiopsids, one sequence; short-branch microsporidians, one sequence. We then tested the primer pairs CM-V5F/CM-V5R, V1F/micuni3R, V1F/530R, V1F/1342R, and V1F/1492R on the sequence dataset. Since primer-template mismatches can hamper a PCR amplification, the number of such mismatches is ideally kept low. We therefore ran several *in silico* PCRs differing in their stringency to identify how well the primers fit the template sequences.

**Figure 1:**
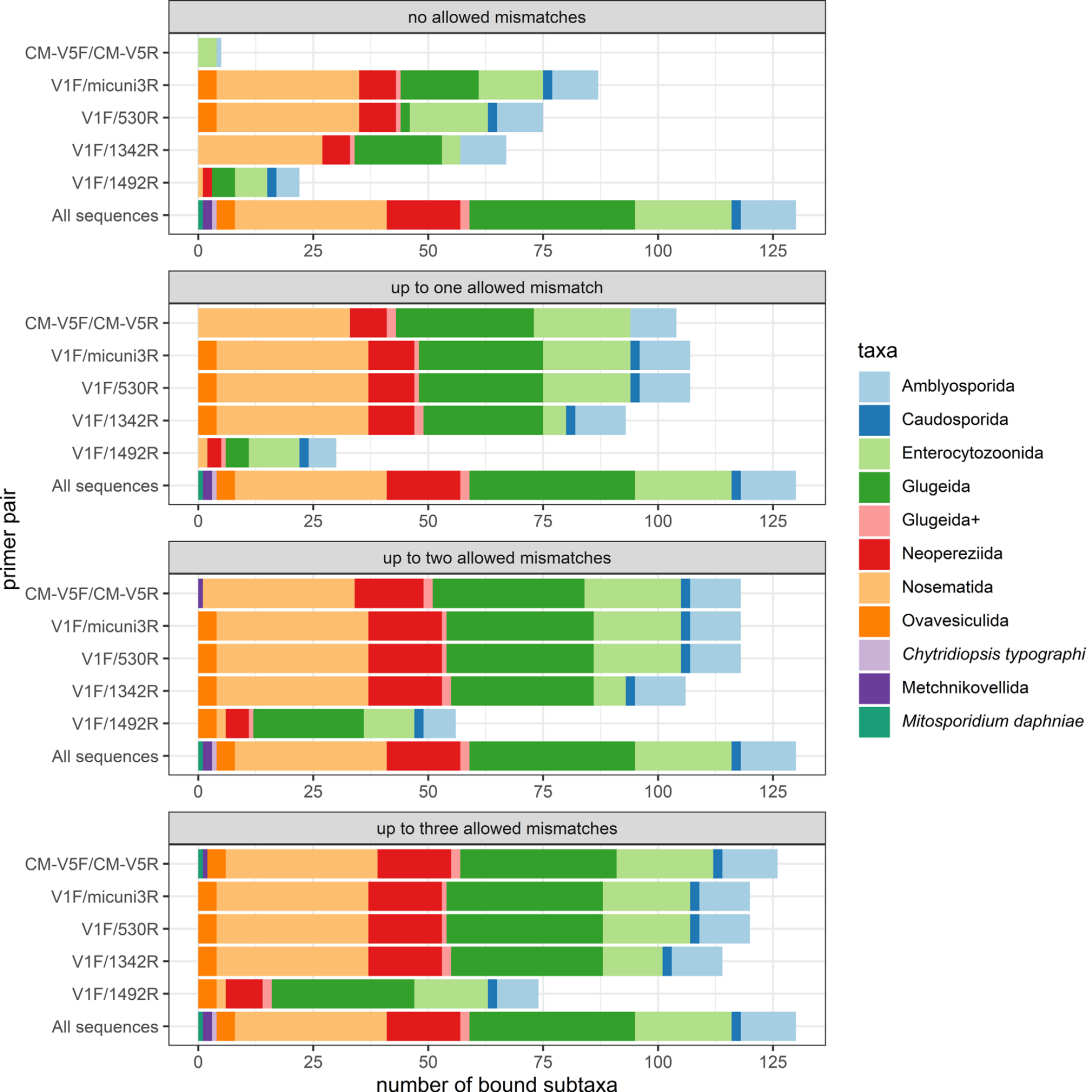
Number of microsporidian species *in silico* amplified by the different primer pair combinations, according to the threshold of accepted mismatches (from none to a maximum of three). The color scheme indicates the corresponding microsporidian subtaxa. The “All sequences”-bar serves as a guidance as it depicts all reference sequences on which the primer pairs were tested.

The overall number of *in silico* amplified microsporidians rose with the number of allowed mismatches for all primer pairs. We found differences in the amplified species number when comparing the primer pairs (Figure 1, Table S3): For example, CM-V5F/CM-V5R amplified the fewest microsporidians without mismatches (3.8% of the sequences used for testing, *n* = 5), whereas V1F/micuni3R amplified the most (66.9%, *n* = 87). When up to three mismatches were tolerated, however, CM-V5F/CM-V5R amplified the most microsporidians out of all primer pairs (96.9%, *n* = 126), with V1F/micuni3R and V1F/530R slightly fewer (92.3%, *n* = 120). The high number of amplified species by CM-V5F/CM-V5R fits the purpose of the primer pair that was specifically developed for metabarcoding of microsporidians [25]. With regard to the two long-read primer pairs, V1F/1342R typically amplified fewer microsporidians than the short-read primer pairs, but performed notably better than V1F/1492R, which amplified the fewest in most PCR settings.

The *in silico* amplicon sizes were comparable to those described in *in vitro* studies. All target amplicons generated by CM-V5F/CM-V5R differed between 153 bp and 225 bp, with a mean of 170.40 bp (compare Figure S1, Table S4). Those of V1F/micuni3R ranged from 393 bp to 474 bp, and were 430.15 bp on average. The amplicon sizes for V1F/530R differed between 389 bp and 470 bp, with a mean of 426.15 bp. All of these short fragment lengths can be sequenced with short-read sequencing platforms. V1F/1342R produced amplicons between 1118 bp and 1348 bp, with an average size of 1209.15 bp. The amplicons predicted for V1F/1492R varied between 1204 bp and 1415 bp, and were 1320.50 bp on average, which requires long-read sequencing platforms. Interestingly, the amplicons show a bimodal size distribution for V1F/micuni3R and V1F/530R (Figure S1), likely due to common deletions occurring in some taxa (compare File S2). These amplicons should therefore appear as agarose gel electrophoresis bands of visibly differing heights more frequently, depending on the microsporidian species present in a sample. A virtual gel, depicting the gel bands for the smallest and largest target amplicon is shown in Figure S2.

All primer pairs remained specific to classical microsporidians and started amplifying representatives of all orders within this subtaxon from a certain mismatch threshold. CM-V5F/CM-V5F additionally amplified one metchnikovellid when we accepted two mismatches, and the short-branched *Mitosporidium daphniae* when setting the threshold to three mismatches (Figure 1). Some primer pairs had difficulties to amplify some classical microsporidian orders: CM-V5F/CM-V5R only started amplifying Ovavesiculida when tolerating up to three mismatches and V1F/1492R missed most of the Nosematida, a highly represented order in the analyzed sequence dataset (Figure 1). We provide more detailed information about the predicted binding events in the supplements (File S2, Table S3).

**Figure 2:**
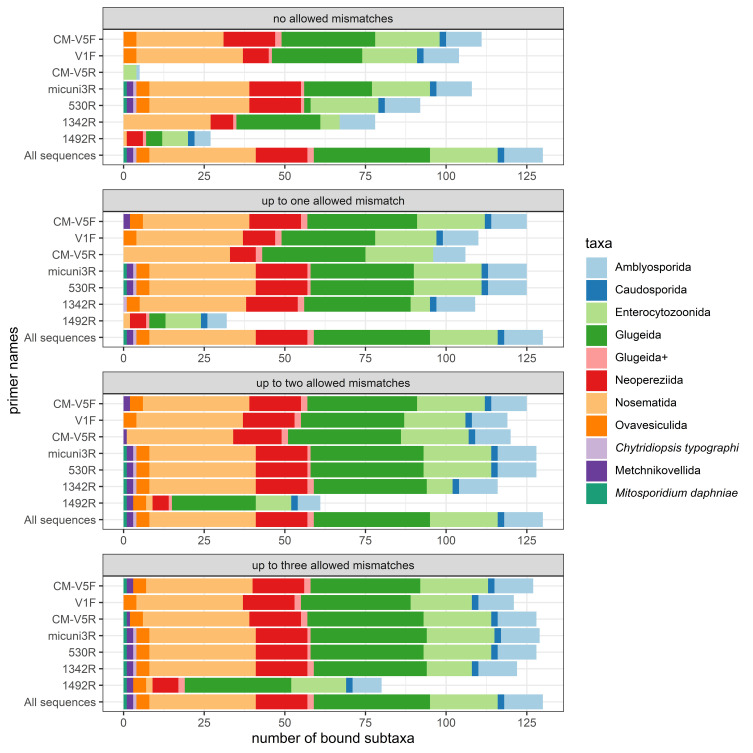
Number of microsporidian species to which each single primer *in silico* bound, according to the threshold of accepted mismatches (from none to a maximum of three). The color scheme indicates the corresponding microsporidian subtaxa. The “All sequences”-bar serves as a guidance and depicts all reference sequences on which the primers were tested.

We then tested each primer individually, as testing primers with the ‘pairs only’ function enabled can obscure what further targets a primer can bind to, if it is not constrained by its primer partner. We found that CM-V5F bound to the most microsporidians without any mismatches (85.4%, *n* = 111; Figure 2), followed by micuni3R (83.1%, *n* = 108) and V1F (80.0%, *n* = 104). By contrast, CM-V5R only bound to five microsporidians (3.8%) in this particular *in silico* test run. We identified a common mismatch between CM-V5R and the sequences likely leading to this observation, which concerns the 6th nucleotide of the primer sequence (File S2, Table S5). During the least stringent *in silico* PCR, micuni3R achieved the highest amplification success with 129 bound microsporidians (99.2%). All other primers showed similar performance, except for 1492R (61.5%, *n* = 80; Figure 2), which generally bound to the fewest microsporidians in most PCRs. 1492R is considered universal as it is based on a highly conserved region of the SSU-rRNA across various organisms including bacteria [16, 29]. Nevertheless, this primer might not be suitable to cover modifications occurring in some microsporidians, especially those that are presumably characteristic for Nosematida.

The only exclusively classical microsporidian-targeted primer was V1F, whereas the other primers started binding to additional taxa at some point (Figure 2). The only primers targeting all non-classical microsporidians without any mismatches, however, were micuni3R and 530R. To our knowledge, no primer pair targeting all microsporidian taxa simultaneously exists so far. These two reverse primers are therefore promising for the development of such a primer pair if a suitable forward primer is found to pair them with.

We also found off-targets for two primers. Such off-target amplifications would often be screened out during size-selected gel cutting. V1F/1492R produced an off-target from *Mockfordia xanthocaeciliae* without mismatches; this off-target, however, was positioned very closely to the actual binding site and likely resulted from several “N”s in the sequence (File S2). V1F/530R started amplifying off-targets from *Gurleya vavrai* and *Paranosema locustae* when three mismatches were accepted. The off-target bindings of the individually tested primers 1492R and 530R corresponded to those already identified in the primer pair testing (for details, see Tables S6 and S7).

It should be noted here that we described *in silico* analyses, but did not consider all factors affecting the outcome of environmental metabarcoding studies out in nature. These factors include the mismatch position (e.g., [6]), the amplification of non-target taxa (e.g., [24]), or adjusting the PCR conditions such as the annealing temperature for improvement (e.g., [12]). The 530R primer, for example, reportedly picks up various non-target taxa such as annelids, apicomplexans, and nematodes [1], also when being combined with V1F [36], whereas Trzebny et al. [25] identified their primer pair CM-V5F/CM-V5R to be more specific compared to V1F/530R. As already demonstrated by Ficetola et al. [10], *in silico* PCR results can differ from those achieved *in vitro* to some extent, but may improve a study as *a priori* primer pair comparisons are being made. Additionally, *in silico* PCRs allow primer testing on a great and self-chosen diversity of target taxa, including rare species or some that would not co-occur in natural samples.

## Conclusion

Our *in silico* analyses indicate slight differences in the observed microsporidian richness inferred from using the tested primer pairs, and that other microsporidian subtaxa besides the classical microsporidians are largely absent in the resulting *in vitro* metabarcoding data. There is a need for designing a primer pair targeting an even broader spectrum of microsporidians, and we identified reverse primers that may give us a good starting point to do so. These two primers, micuni3R [28] and 530R [2], may be useful for developing new primer pair combinations suitable for short-read sequencing platforms. It would also be useful to develop better primer pairs for long-read sequencing that amplify Microsporidia *sensu lato*.

## Supplementary material

The supplementary material of this article is available at https://www.parasite-journal.org/10.1051/parasite/2023056/olm. *Figure S1:* Sizes of predicted amplicons according to primer pair. Colors indicate the threshold for the number of accepted mismatches in the *in silico* PCR run. Off-target amplicons were removed from this figure.
*Figure S2:* Virtual gel depicting the smallest and largest *in silico* predicted target amplicon for each primer pair (maximum number of allowed mismatches: 3). Image was created with the Virtual Gel function in Geneious (Biomatters) using the MW Ladder: 100 bp (NEB).
*File S1:* Reduced version of the alignment originally published in Dubuffet et al. [9], only including sequences that we selected for the *in silico* analyses.
*File S2:* Visualization of the reduced alignment originally published in Dubuffet et al. [9] showing the primer bindings. The image was created in Geneious (Biomatters) with the maximum of allowed mismatches set to three. Note that some off-targets appear due to longer sequence regions consisting of “N”s which did not appear in the actual analyses and were therefore interpreted as erroneous.
*Table S1:* Selection of example literature in which the analyzed primer pairs were used.
*Table S2:* Overview of the analyzed sequences that were selected from an alignment published in Dubuffet et al. [9].
*Table S3:* Number of amplified species within each clade, according to primer pair and number of allowed mismatches in the corresponding *in silico* PCR run. Off-targets are excluded to avoid multiple counting of the same microsporidian.
*Table S4:* Amplicon sizes according to primer pair and number of accepted mismatches in the corresponding *in silico* PCR run.
*Table S5:* Identified mismatch combinations for each individual primer tested.
*Table S6:* Overview of off-target amplicons identified in the *in silico* PCR runs for primer pairs only.
*Table S7:* Overview of off-target bindings identified in the *in silico* PCR runs for each individual primer.
