# Supplementary figures and images for: Comparing Microsporidia-targeting primers for environmental DNA sequencing
Source: Parasite. 2023 Nov 28;30:52. doi: 10.1051/parasite/2023056 (PMC10683580; doi:10.1051/parasite/2023056)

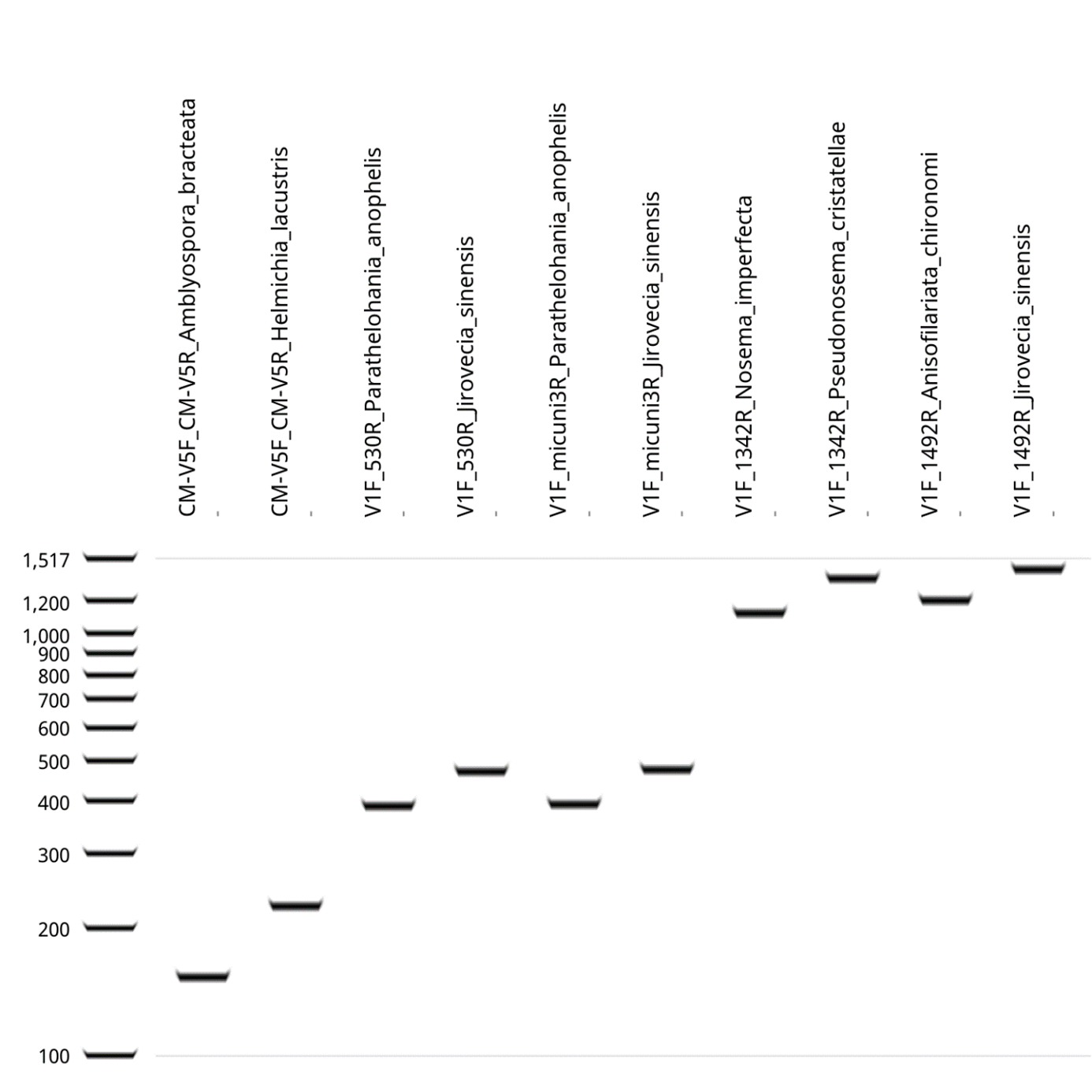

Supplement: Supplementary file 1 — Figure S1: Sizes of predicted amplicons according to primer pair. Colors indicate the threshold for the number of accepted mismatches in the in silico PCR run. Off-target amplicons were removed from this figure. Figure S2: Virtual gel depicting the smallest and largest in silico predicted target amplicon for each primer pair (maximum number of allowed mismatches: 3). Image was created with the Virtual Gel function in Geneious (Biomatters) using the MW Ladder: 100 bp (NEB). File S1: Reduced version of the alignment originally published in Dubuffet et al. [9], only including sequences that we selected for the in silico analyses. File S2: Visualization of the reduced alignment originally published in Dubuffet et al. [9] showing the primer bindings. The image was created in Geneious (Biomatters) with the maximum of allowed mismatches set to three. Note that some off-targets appear due to longer sequence regions consisting of “N”s which did not appear in the actual analyses and were therefore interpreted as erroneous. Table S1: Selection of example literature in which the analyzed primer pairs were used. Table S2: Overview of the analyzed sequences that were selected from an alignment published in Dubuffet et al. [9]. Table S3: Number of amplified species within each clade, according to primer pair and number of allowed mismatches in the corresponding in silico PCR run. Off-targets are excluded to avoid multiple counting of the same microsporidian. Table S4: Amplicon sizes according to primer pair and number of accepted mismatches in the corresponding in silico PCR run. Table S5: Identified mismatch combinations for each individual primer tested. Table S6: Overview of off-target amplicons identified in the in silico PCR runs for primer pairs only. Table S7: Overview of off-target bindings identified in the in silico PCR runs for each individual primer. [file parasite-30-52-s1.zip › FileSuppS2.png]
